# Effects of combined training or moderate intensity continuous training during a 3-week multidisciplinary body weight reduction program on cardiorespiratory fitness, body composition, and substrate oxidation rate in adolescents with obesity

**DOI:** 10.1038/s41598-023-44953-3

**Published:** 2023-10-17

**Authors:** Mattia D’Alleva, Stefano Lazzer, Gabriella Tringali, Roberta De Micheli, Adele Bondesan, Laura Abbruzzese, Alessandro Sartorio

**Affiliations:** 1 Department of Medicine, University of Udine, P.le Kolbe 4, 33100 Udine, Italy; 2 School of Sport Science, University of Udine, Udine, Italy; 3 Experimental Laboratory for Auxo-endocrinological Research, Istituto Auxologico Italiano, IRCCS, Piancavallo-Verbania, Italy; 4 Experimental Laboratory for Auxo-endocrinological Research, Istituto Auxologico Italiano, IRCCS, Piancavallo-Verbania, Italy; 5 Experimental Laboratory for Auxo-endocrinological Research, Istituto Auxologico Italiano, IRCCS, Milan, Italy

**Keywords:** Metabolism, Health care

## Abstract

This study aimed to investigate the effects of combined training (COMB, a combination of moderate-intensity continuous training-MICT and high-intensity interval training-HIIT) *vs.* continuous MICT administered during a 3-week in-hospital body weight reduction program (BWRP) on body composition, physical capacities, and substrate oxidation in adolescents with obesity. The 3-week in-hospital BWRP entailed moderate energy restriction, nutritional education, psychological counseling, and two different protocols of physical exercise. Twenty-one male adolescents with obesity (mean age: 16.1 ± 1.5 years; mean body mass index [BMI] 37.8 ± 4.5 kg m^−2^) participated in this randomized control trial study (n:10 for COMB, n:11 MICT), attending ~ 30 training sessions. The COMB group performed 3 repetitions of 2 min at 95% of peak oxygen uptake (V′O_2_ peak) (e.g., HIIT ≤ 20%), followed by 30 min at 60% of V′O_2_ peak (e.g., MICT ≥ 80%). Body composition, V′O_2_ peak, basal metabolic rate (BMR), energy expenditure, and substrate oxidation rate were measured during the first week (W0) and at the end of three weeks of training (W3). The two training programs were equivalent in caloric expenditure. At W3, body mass (BM) and fat mass (FM) decreased significantly in both groups, although the decrease in BM was significantly greater in the MICT group than in the COMB group (BM: − 5.0 ± 1.2 *vs*. − 8.4 ± 1.5, P < 0.05; FM: − 4.3 ± 3.0 *vs*. − 4.2 ± 1.9 kg, P < 0.05). V′O_2_ peak increased only in the COMB by a mean of 0.28 ± 0.22 L min^−1^ (P < 0.05). The maximal fat oxidation rate (MFO) increased only in the COMB group by 0.04 ± 0.03 g min^−1^ (P < 0.05). COMB training represents a viable alternative to MICT for improving anthropometric characteristics, physical capacities, and MFO in adolescents with obesity during a 3-week in-hospital BWRP.

## Introduction

Obesity is a global health problem affecting millions of people worldwide, especially children and adolescents^1^. According to the World Health Organization (WHO), the prevalence of obesity in adolescents aged 10–19 years has increased from 4% in 1975 to 18% in 2016^2^. The main causes of obesity are a sedentary lifestyle, unhealthy diet, and lack of reduced physical activity level with negative consequences on physical and mental health, such as the increased risk of cardiovascular disease, diabetes, hypertension, dyslipidemia, inflammation, and psychosocial complications^3^. Indeed, compared with lean counterparts, individuals with obesity exhibit exercise intolerance, lower cardiorespiratory fitness (CRF)^4^, and impaired capacity to oxidize lipids at rest^5^ and during physical activity^6^ which may compromise their current and future health^7^. CRF is one of the most important indicators of health and fitness (i.e., typically expressed as maximal oxygen uptake, V′O_2_ max, or peak oxygen uptake, V′O_2_ peak), reflecting the ability of the heart and lungs to deliver oxygen to the muscles during exercise^8^. CRF is inversely related to mortality and morbidity in various chronic diseases^9^. Therefore, aerobic training is critical in weight management programs for adolescents with obesity to create an energy deficit that reduces body mass (BM) and fat mass (FM)^10^, improves CRF^11^, and optimizes substrate oxidation capacity.

Two types of exercise training have received much attention in recent years: moderate-intensity continuous training (MICT) and high-intensity interval training (HIIT). MICT consists of performing aerobic exercise at a constant and moderate intensity for a prolonged duration (i.e., 30–50 min at ≤ 80% of maximal heart rate, HRmax)^12^, whereas HIIT consists of alternating short periods of intense exercise (i.e., duration between 1 and 4 min at ≥ 85% of HRmax)^13,14^ with low-intensity exercise recovery periods with a total duration between 4 and 16 min. Both MICT and HIIT have been shown to improve body composition^15,16^, CRF^12,15^, and metabolic health^12,15^ in adolescents with obesity, although HIIT is a time-efficient form of exercise^15^. However, a previous meta-analysis reported the occurrence of adverse events such as leg discomfort, joint sprains, and asthma during and after HIIT^17^. Furthermore, given the heterogeneity and low number of studies comparing the HIIT and MICT protocols, there is limited evidence on the comparative effects of MICT and HIIT in adolescents with obesity^18^.

Analysis data from previous studies found that the combination of MICT (i.e., 70–80% of total training volume, 30–40 min session^−1^ at 65–70% of HRmax or 90% of the first ventilatory threshold) and HIIT (i.e., ~ 20–30% of total training volume, 6–12 min session^−1^ at 80–90% of HRmax or 90% of V′O_2_peak)^19–21^ with a polarized approach^22^ performed in the same training session or a weekly training program resulted in equal or greater effects on CRF, body composition^19–21^ and substrate oxidation^20,21,23^ in lean and obese sedentary adults compared with MICT or HIIT alone with equal volume or energy expenditure per session. Since people with obesity have reduced cardiorespiratory fitness^9^, reduced mitochondrial function and poor metabolic flexibility^24^, combining HIIT and MICT with a polarized approach would be an effective strategy to improve both mitochondrial content and mitochondrial functionality compared to MICT alone^25^. Furthermore, to the best of our knowledge, no study in adolescents with obesity has used a priori percentage of a combination of MICT and HIIT with a polarized approach during weekly training to improve cardiorespiratory function and substrate oxidation rate compared with MICT alone. To optimize cardiorespiratory function and substrate oxidation during moderate or interval training, several authors recommended walking or running instead of cycling^26,27^.

Therefore, the present study aimed to evaluate the effects of a 3-week combination of HIIT and MICT (combined training; COMB) using a polarized approach^21^ and MICT alone on: body composition, V′O_2_ peak, and substrate oxidation rates in adolescents with obesity hospitalized for a 3-week multidisciplinary body weight reduction program (BWRP), entailing moderate energy restriction, nutritional education, psychological counseling (the same for all participants). We hypothesized that COMB would be more effective than MICT in improving these outcomes in adolescents with obesity.

## Results

### Anthropometric characteristics and body composition

At W0, no significant differences were found between the two groups for age, BM, BMI, FFM, and FM (kg). FM (%) was higher in COMB than in the MICT group (44.0 ± 4.6 *vs*. 37.8 ± 2.1%, group effect, P = 0.003, Table 1). BMR (MJ die^−1^) and BMR (MJ kg FFM^−1^ die^−1^) were lower in COMB than in the MICT group (7.44 ± 1.05 *vs*. 8.66 ± 0.78 MJ die^−1^, group effect P = 0.023, and 0.11 ± 0.01 *vs*. 0.13 ± 0.02 MJ kg FFM^−1^ die^−1^, group effect P = 0.019, Table 1), respectively.

**Table 1:** Anthropometric characteristics and physical capacities of adolescents before (Week 0) and at the end (Week 3) of the multidisciplinary weight-management program in combined training (COMB) and moderate-intensity continuous training (MICT) groups.

	COMB (n: 10)		MICT (n: 11)		P		
	Week 0	Week 3	Week 0	Week 3	G	T	G × T
Age (years)	15.7 ± 1.7	15.8 ± 1.7	16.2 ± 1.1	16.3 ± 1.1	0.309	0.001	0.835
Stature (m)	1.75 ± 0.07	1.75 ± 0.07	1.72 ± 0.07	1.72 ± 0.07	0.667	0.165	0.230
Body mass (kg)	117.7 ± 19.8	112.6 ± 19.2*	111.5 ± 14.8	103.2 ± 13.9*	0.340	0.001	0.001
BMI (kg m^−2^)	38.5 ± 5.3	36.9 ± 5.2*	37.1 ± 3.1	34.3 ± 3.0*	0.335	0.001	0.001
Fat-free mass (kg)	65.4 ± 7.9	64.7 ± 7.5	69.2 ± 7.5	65.0 ± 8.4*	0.567	0.001	0.001
Fat Mass (kg)	52.3 ± 13.2	48.0 ± 12.8*	42.4 ± 7.6	38.2 ± 6.0*	0.060	0.001	0.889
Fat Mass (%)	44.0 ± 4.6	42.0 ± 4.8*	37.8 ± 2.1	37.0 ± 2.0	0.003	0.009	0.268
BMR (MJ die^−1^)	7.44 ± 1.05	7.20 ± 1.02	8.66 ± 0.78	8.13 ± 1.02*	0.022	0.012	0.286
BMR (MJ kg FFM^−1^ die^−1^)	0.11 ± 0.01	0.11 ± 0.01	0.13 ± 0.02	0.13 ± 0.01	0.018	0.564	0.868
V′O_2_peak (L min^−1^)	2.52 ± 0.46*	2.81 ± 0.45*	3.60 ± 0.42	3.62 ± 0.36	0.001	0.014	0.035
V′O_2_peak (mL min^−1^ kg FFM^−1^)	38.6 ± 5.3	43.3 ± 4.6*	52.2 ± 5.3	56.3 ± 7.5	0.003	0.001	0.773
HRpeak (bpm)	176.0 ± 4.7	176.0 ± 9.1	176.1 ± 6.8	177.8 ± 5.6	0.668	0.609	0.572
O_2_pulse (ml bpm^−1^)	14.3 ± 2.5	15.9 ± 2.5*	20.4 ± 2.3	20.4 ± 2.3	0.001	0.040	0.038

All values are presented as mean ± standard deviation.

BMI, body mass index; BMR, basal metabolic rate; V′O_2_max, maximal oxygen uptake; HRmax, heart rate max; O_2_; oxygen.

G, group effect; T, time effect; G×T, groups × time effect.

*Significantly different from W0, P < 0.05.

Between W0 and W3, V′O_2_peak (L min^−1^) and O_2_pulse increased significantly only in the COMB group by + 0.28 ± 0.22 L min^−1^ (ES: 0.61, *large*, interaction G × T P = 0.035) and + 1.62 ± 1.53 ml bpm^−1^ (ES: 0.62, *large*, interaction G × T P = 0.038). V′O_2_peak expressed in relative values significant increase in the COMB (by 4.76 ± 4.28 ml kg FFM^−1^ min^−1^, ES: 0.90, *large*, time effect P < 0.001) and MICT (by 4.15 ± 4.87 ml kg FFM^−1^ min^−1^, ES: 0.61, *large*, time effect P < 0.001), groups. However, HRpeak did not change significantly in both groups (main effect of time, P = 0.609) (Table 1).

### Substrate oxidation rate during the graded test

**Figure 1:**
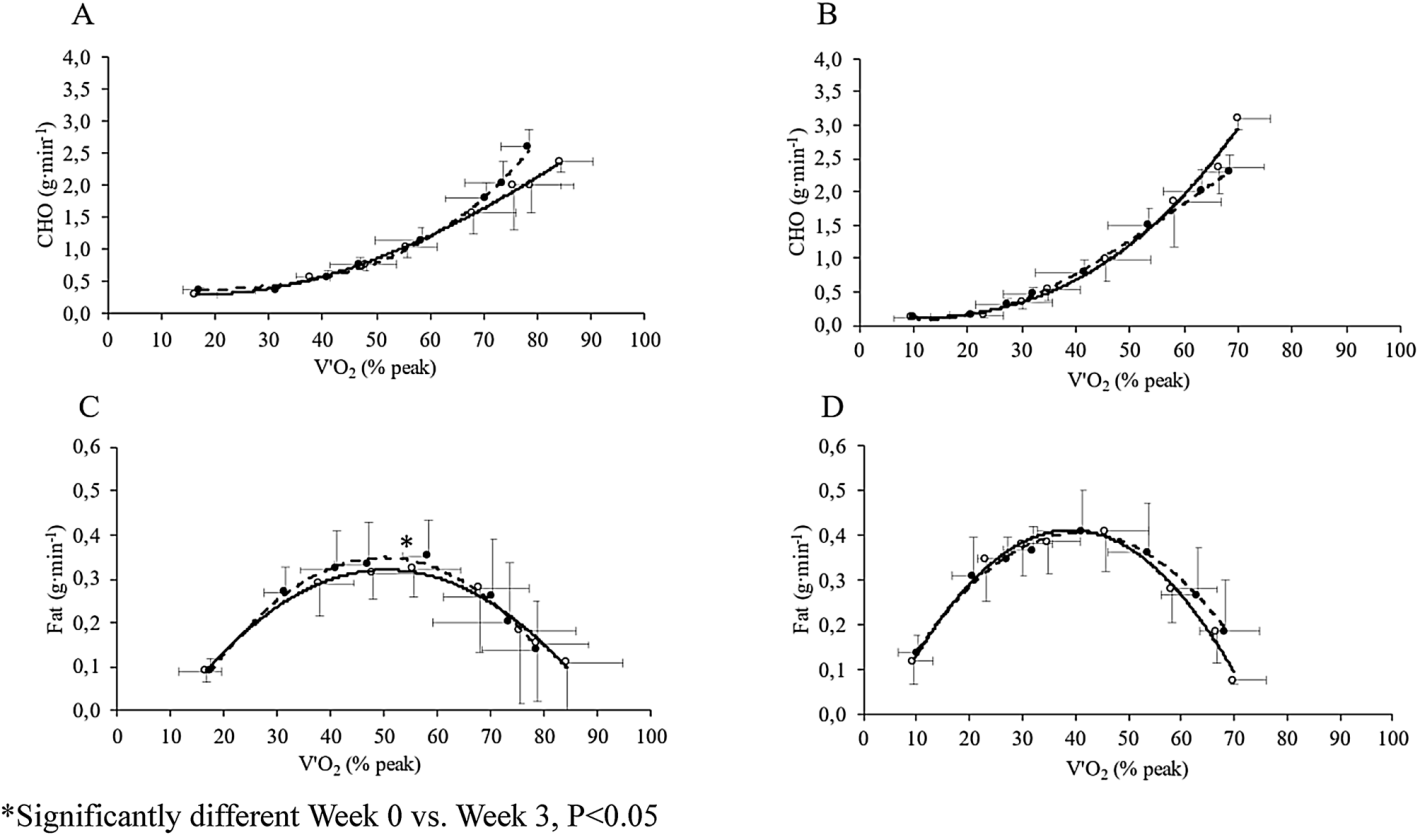
Carbohydrate (CHO, g min^−1^, panels **A** and **B**) and fat (g min^−1^, panels **C** and **D**) oxidation rates as a function of exercise intensity expressed as percent of peak oxygen uptake (V′O_2_peak) in COMB (panels **A** and **C**) and MICT (panels **B** and **D**) groups, before (Week 0, opened circle and continuous line) and at the end (Week 3, filled circle and dashed line) of the multidisciplinary weight-management program. *Significantly different Week 0 vs. Week 3, P < 0.05.

At baseline, CHO oxidation rates were not significantly different between the COMB and MICT (main effect of group, P = 0.060) during the graded test at all exercise intensities (Fig. 1, panels A and B). Moreover, the CHO oxidation rate, expressed in absolute values, increased with exercise intensity in both groups (Fig. 1, panels A and B). Fat oxidation rates were lower in the COMB group than in the MICT group at exercise intensities above 35 ± 6% of V′O_2_peak (main effect of group P = 0.009). The maximal fat oxidation rate (MFO) rate was observed at 50 ± 9% of the V′O_2_ peak in the COMB group (0.32 ± 0.07 g min^−1^, Fig. 1 panel C) and at 46 ± 8% of the V′O_2_ peak in the MICT group (0.41 ± 0.09 g min^−1^, Fig. 1 panel D). The MFO, expressed in absolute values, was lower in the COMB group than in the MICT group (0.32 ± 0.06 vs. 0.41 ± 0.09 g min^−1^, respectively, P < 0.001). At exercise intensities above 66 ± 12% of the V’O_2_ peak, the fat oxidation rate decreased markedly in both groups, and the contribution of fat oxidation to the energy supply became negligible above 74 ± 6% of the V′O_2_ peak.

At POST, CHO and fat oxidation rates at all exercise intensities in the COMB and MICT groups were not significantly different from those at W0 (Fig. 1 panels A–D). Exercise intensity corresponding to the MFO rate, expressed in absolute value increased only in the COMB group by 0.04 ± 0.03 g min^−1^ (ES. 0.38, *medium*, P < 0.001) (Fig. 1 panel C and D).

### Training characteristics

Participants in both groups completed 28 ± 2 sessions of physical exercise. On average, each exercise session lasted less time in the COMB group (35.9 ± 4.6 min) than in the MICT group (45 ± 6 min, P < 0.001). At W0, the average HR during the training sessions was 134 ± 7 bpm and 124 ± 6 bpm in the COMB and MICT groups, respectively (P < 0.010). HR expressed in relative values, was higher in the COMB group (76 ± 3%HRmax), than in the MICT group (70 ± 3%HRmax, group effect P < 0.001).

At W3, HR expressed in absolute values did not change in both groups (time effect P = 0.147 and P = 0.170, respectively). On average, the COMB group performed 17.0 ± 1.9% of the weekly training volume at HIIT and 83.1 ± 1.9% at MICT, whereas the MICT group spent 100% of the weekly training volume at MICT.

### Energy expenditure and substrate oxidation rate during the submaximal exercise

At W0, energy expenditures (EEs) were not significantly different during the COMB and MICT exercise (1362 ± 166 *vs*. 1521 ± 256 kJ, respectively, P = 0.066). During the exercise, both groups had similar energy from CHO and protein (Table 2). The amount of fat oxidized during exercise was significantly lower in the COMB group than in the MICT group (552 ± 137 *vs*. 831 ± 177 kJ, group effect P = 0.002) (Table 2).

**Table 2:** Substrate oxidized during Combined training (COMB) and Moderate-intensity continuous training (MICT) exercises before (Week 0) and at the end (Week 3) of the multidisciplinary body weight-management program.

	COMB (n:10)		MICT (n: 11)		P		
	Week 0	Week 3	Week 0	Week 3	G	T	G × T
Total EE (kJ)	1362 ± 166	1312 ± 184	1521 ± 256	1549 ± 201	0.070	0.912	0.410
EE from CHO (kJ)	644 ± 189	737 ± 211	518 ± 107	563 ± 142	0.117	0.159	0.592
EE from Fat (kJ)	552 ± 137	433 ± 157	831 ± 178	812 ± 221	0.002	0.091	0.168
EE from Protein (kJ)	174 ± 20	165 ± 22	172 ± 30	173 ± 22	0.686	0.541	0.370
CHO (g)	39 ± 11	44 ± 13	31 ± 6	34 ± 9	0.114	0.162	0.592
Fat (g)	15 ± 4	11 ± 5	22 ± 5	22 ± 6	0.002	0.092	0.169
Protein (g)	10 ± 1	10 ± 1	10 ± 2	10 ± 1	0.710	0.532	0.364

All values are presented as mean ± standard deviation.

EE, energy expenditure; CHO, carbohydrate.

G, group effect; T, time effect; G×T, groups × time effect.

*Significantly different from W0, P < 0.05.

At W3, EEs, CHO, fat, and protein oxidation rates did not change significantly in the COMB and MICT groups (Table 2).

## Discussion

The present study showed that 3-week in-hospital multidisciplinary BWRP with COMB or a MICT training program in adolescents with obesity (1) significantly reduced BM and FM in both groups, although more pronounced in the MICT group; (2) maintained FFM only in the COMB group; (3) significantly improved V′O_2_peak and O_2_ pulse in the COMB group; and (4) determined an increase in MFO during the GRAD test only in the COMB group.

The first important finding was that both COMB and the MICT program helped to reduce BM and FM, by ~ 5 and 8 kg for BM and ~ 4 kg in both groups for FM. Although MICT was found to be more effective in reducing BM, both types of training contributed equally to reducing FM. A recent meta-analysis showed that both MICT and HIIT produced similar reductions in BM (i.e., between 2 and 5 kg) kg in adolescents with obesity over an average period of 12 weeks^12,28^. In our study, we observed that a 3-week multidisciplinary BWRP entailing moderate energy restriction, nutritional education, psychological counseling (for all participants), and two different training methods (i.e., COMB and MICT) was capable of determining a similar weight loss in one-third of the time compared with previous data^12,28^. Nevertheless, participants allocated to the COMB or MICT group received the same balanced diets formulated according to the Italian recommended dietary allowances^29^, it is very difficult to determine the extent to which the different components of the training study such as moderate energy restriction, physical exercise, nutritional education, psychological counseling and the growth process of adolescence explain the reduction in BM and FM. Since most cases of obesity in childhood and adolescence are caused by insufficient physical activity^30^, it is important to reduce BM and FM at a young age with a specific program that includes physical training combined with energy restriction in a specialized institution to (1) reduce the risk of developing obesity in adulthood^31^ and (2) reduce the prevalence of obesity-related diseases such as type 2 diabetes, stroke, coronary heart disease, and cancer^32^. From a physiological perspective, COMB and MICT helped in reducing BM and FM may be due to similar improvements in skeletal muscle capacity to increase the glycogen and fatty acid content and utilization during aerobic exercise^33,34^ which are typically impaired in obesity^24^. In the current study, we observed higher fat oxidation (i.e., 41–54%) and lower carbohydrate oxidation (i.e., 35–47%) during submaximal exercise in both COMB and MICT groups. Thus, to the best of our knowledge, this study is the first to use COMB training with a polarized approach in a cohort of adolescents with obesity to reduce BM and FM. In addition, FFM was maintained only in the COMB group, whereas a large BM loss in the MICT group consisted mainly of FFM. The within-group comparison showed that BMR decreased significantly in the MICT group (-6%, ES: 056, *large*) compared with the COMB group (-2%, ES: 0.21, *medium*). Recent evidence has shown that HIIT can promote the anabolic pathway leading to increased muscle protein synthesis and muscle satellite cell activation in athletes and clinical patients^35^, and the COMB group performed a small amount of HIIT (i.e., ~ 16% of total weekly volume), compared to the MICT group.

The second important finding was that COMB training significantly increased V′O_2_ peak in absolute and relative values by ~ 12%, confirming previous results observed in adults with obesity. Indeed, previous studies have shown that a combination of high-volume, low-intensity exercise (i.e., 80% of the overall training volume) and low-volume, high-intensity training (i.e., < 20%)^20,21,36^ resulted in equal or better improvements in V′O_2_ peak compared to HIIT or MICT alone (i.e., improvements ranging between 15 and 25%) in adults with obesity and in highly trained endurance athletes^21,36,37^. Compared to the above-mentioned studies, our study presents two important differences (1) it is the first study using COMB training with a polarized approach in a group of adolescents with obesity, (2) it was conducted in only 3 weeks, compared to the average of 12–16 weeks of previous studies. Moreover, the results obtained in our study confirmed that the improvements obtained by COMB were similar to those obtained by HIIT (i.e., + 12% V′O_2_peak) in a group of adolescents with obesity^16^. However, no study to date has compared the effects of COMB and HIIT on V′O_2_peak in a group of adolescents with obesity. The effect of COMB on improving V′O_2_peak may be better than that of MICT, due to an increase in factors affecting oxygen delivery and extraction, including stroke volume (i.e., confirmed by an increase in O_2_ pulse as an indirect marker of central adaptations)^38^, peripheral perfusion and diffusing capacity and skeletal muscle oxidative capacity^25^. Nonetheless, we observed positive results for the first time with an a priori manipulation of HIIT and MICT (i.e., ~ 85% MICT and ~ 15% HIIT) in the COMB group when applied to adolescents with obesity.

The third finding was that the increase in MFO occurred only in the COMB group (i.e., + 0.04 g min^−1^, + 6%), with no significant differences for CHO and fat oxidation at all exercise intensities in the COMB and MICT groups. Our study is consistent with current scientific literature, as HIIT and SIT, the most effective types of exercise for improving fat oxidation, produce a mean increase in fat oxidation of ~ 0.03 g min^−1^ when applied over a short period of time (i.e., < 4 weeks)^39^. Previous studies conducted in adults with obesity showed that MFO increased after a period of MICT (i.e., intensity > 70% of V′O_2_peak) with or without significant weight loss^40,41^. However, in our study the MICT group showed a large BM decrease compared to the COMB group, even MFO remained unchanged. There may be a minimum intensity to observe an increase in the absolute value of MFO. In our study, the MICT group trained at an intensity of ~ 40% of V′O_2_peak, compared with 60% of V′O_2_peak in the MICT portion of the COMB group, despite different training volumes. A previous study by our research group showed that HIIT increased fat oxidation more than MICT alone in adolescents with obesity^16^. Consistent with a recent meta-analysis^39^, our study showed that the combination of low-volume HIIT and moderate-volume exercise (i.e., COMB) is effective in improving fat oxidation, metabolic health, and body composition in individuals with obesity^39^. The key mechanisms underlying these metabolic adaptations induced by COMB training appear to be mediated primarily in type I fibers^42^, which have high intramuscular fatty acid oxidation rates and high mitochondrial content^25,43^.

The present study has several limitations. First, although we have reported that 3 weeks of COMB or MICT training combined with moderate energy restriction and nutrition education improves body composition and physical performance, it is difficult to determine the determining factor for body composition improvement because we did not have an inactive control group to rule out the dietary factors. Second, since the recruited boys are still growing, we do not know to what extent this factor was decisive for the improvement in body composition and physical capacities. Third, in the present study we did not measured the amount of energy spent in aerobic leisure activity. Therefore, we are unable to know what extent this might have had an impact on improving body composition and physical capacities in our study population. Fourth our study was conducted on adolescents with obesity in a specialized institution; therefore, it is not possible to generalize our results to home-based training. Finally, because we compared a priori only one COMB training with MICT, it is difficult to assess whether the combination ratio we chose was indeed optimal or whether there are even better combinations.

In summary, COMB and MICT training helped in improving body composition. In contrast, only the COMB group improved cardiovascular parameters and MFO. Thus, COMB training could be a reasonable alternative to MICT or HIIT to improve body composition and physical capacities in adolescents with obesity. Future studies should examine different combinations of HIIT and MICT (i.e., polarized, pyramidal, or threshold training) over a longer period of time (12 weeks) and compare them with HIIT and MICT alone to determine the optimal combination of HIIT and MICT.

## Material and methods

### Participants

Twenty-one boys with severe obesity (body mass index standard deviation score, BMI SDS > 2, 16.0 ± 1.4 years)^44^ with a pubertal stage > 3^45^ were recruited as inpatients from the Division of Auxology, Italian Institute for Auxology, IRCCS, Piancavallo (VB), Italy. Their BM was stable (changes less than ± 1 kg) during the previous 2 months. Inclusion criteria were i. absence of cardiovascular, respiratory, neurological, musculoskeletal, metabolic, and/or endocrine disease, ii. no regular use of medications known to influence energy metabolism. The 3-week multidisciplinary BWRP included moderate energy restriction, nutritional education, psychological counseling, and two different training programs (COMB or MICT, described in detail in the following paragraph).

### Study protocol

The study was approved by the Ethics Committee of the Istituto Auxologico Italiano, IRCCS, Milan, Italy (ethical committee code of approval: 2022_03_15_03; research project code: 01C212; acronym: ALPOLAROB). The study was conducted in accordance with the Declaration of Helsinki and with the 2005 Additional Protocol to the European Convention of Human Rights and Medicine concerning Biomedical Research. Before the study began, all the volunteers and their parents were fully informed of the purpose of the study and provided written informed consent. The adolescents were hospitalized for a multidisciplinary BWRP. During the first few days, all the participants underwent a physical examination including hematology, biochemistry, and urine analysis. Physical examination included assessment of anthropometric characteristics, body composition, basal metabolic rate (BMR), energy expenditure (EE), and substrate oxidation rate during submaximal exercise. Thereafter, all volunteers followed a 3-week personalized weight-management program entailing moderate energy restriction, nutritional education, and psychological counseling. Participants were randomly allocated (using sealed envelopes and a 1:1 ratio) into two groups: the COMB (n:10) and MICT groups (n:11). All the testing sessions were conducted just before the beginning (week 0, W0) and at the end of the 3-week body weight reduction program (week 3, W3).

### Diet and nutritional education

Based on the initial BMR and physical activity level, all adolescents received a personalized diet during the 3-week BWRP. Energy intake was adjusted to be close to 1.2 times the initial BMR, which is approximately 15–20% lower than the estimated daily EE. Diet composition was formulated according to the Italian recommended daily allowances^29^. During the 3-week weight reduction program, the consumption of meals was always under the supervision of a dietician.

### Physical activity

The training program included two training sessions per day (i.e., from Monday to Friday) for a period of 3 weeks under medical supervision. Volunteers walked on a treadmill. Each participant monitored his intensity with a heart rate (HR) chest strap (Polar H10, Polar Electro Oy, Finland). All subjects completed 28 ± 2 sessions of physical training.

**Figure 2:**
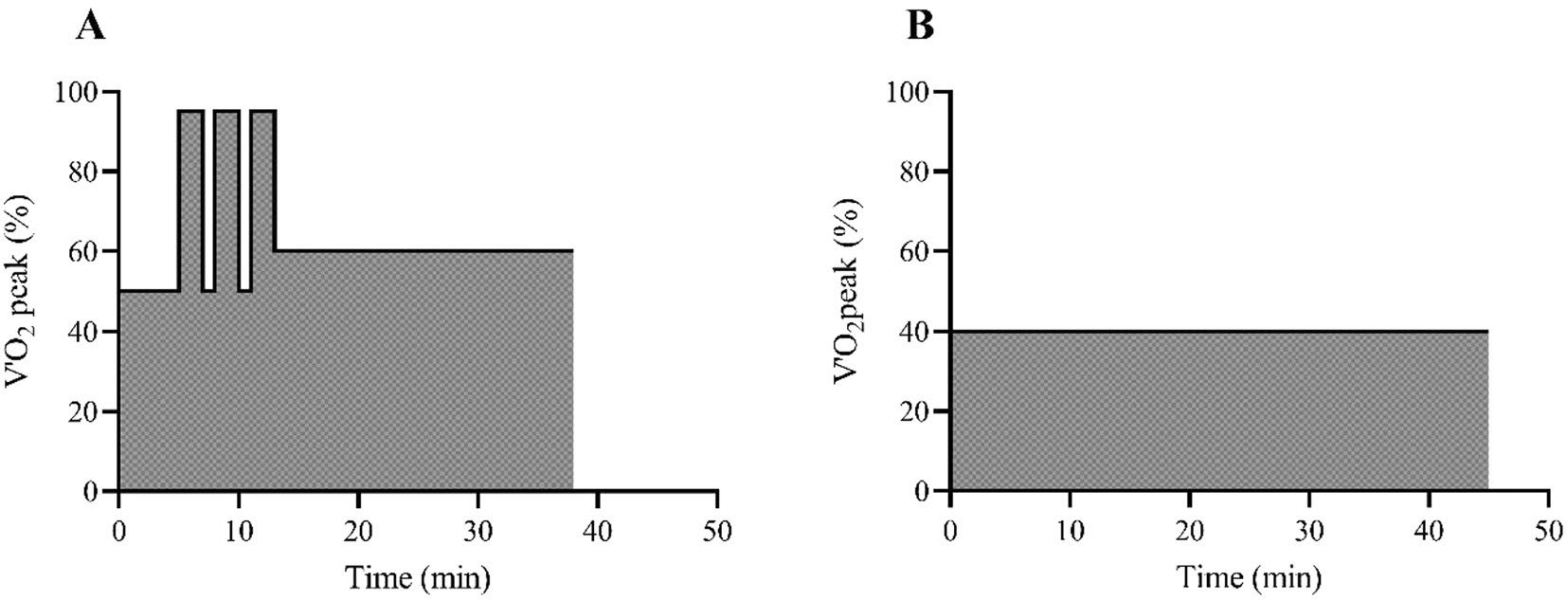
Schematic representation of training protocols: combined training (COMB, panel **A**) and Moderate-intensity continuous training (MICT, panel **B**).

The COMB group completed a combination of high-volume, low-intensity exercise (~ 80% of overall training volume) and low-volume, high-intensity exercise (~ 20%). Each session consisted of a 5-min warm-up (50% of V′O_2_ peak) followed by three 2-min at high intensity (95% of V’O_2_ peak), separated by 1 min of walking at low intensity (50% of V′O_2_ peak), followed by ~ 30 min of MICT (60% of V′O_2_ peak)^21^ (Fig. 2A). Each training session lasted 36 ± 4 min for the COMB program. The MICT group performed 45 ± 6 min at HR corresponding to 40% of V′O_2_peak^16^ (Fig. 2B). Exercise intensity was manipulated by adjusting treadmill speed and incline according to the values of 40, 50, 60, and 95% of V’O_2_ peak measured during the graded test (GRAD) and then calculating the corresponding HR values. Both training programs were equated to the same energy expenditure during a training session (e.g., 20 kJ per kg of fat-free mass, FFM, about 1.4 MJ per session)^16^.

In addition, volunteers had 1 h day^−1^ of aerobic leisure activities at the institution on Saturday and Sunday. The research assistant and physical trainers monitored all of the adolescents’ training sessions to increase participant motivation^46^ and to verify that each subject participated in each training session, performed the exercises correctly, and completed at least 95% of the training program.

### Measurements

#### Physical characteristics and body composition

BM was measured to the nearest 0.1 kg using an electronic scale (Selus, Italy) with the volunteers dressed only in light underwear and no shoes. Stature was measured to the nearest 0.5 cm on a standardized Harpenden stadiometer (Holtain Ltd, UK). The body mass index (BMI) was calculated as BM (kg) × stature (m)^−2^. For body composition analysis, a tetrapolar multifrequency impedance mete impedancemeter (BIA, Human-IM Scan, DS-Medigroup, Milan, Italy) was used after subjects had rested supine for 20 min with arms and legs relaxed and not in contact with other body parts^47^. A current of 800 µA was delivered at a frequency of 50 kHz for the BIA measurements. Great care was taken to standardize the variables affecting the validity, reproducibility, and precision of the measurement. FFM was calculated using the equation developed by Lazzer et al.^48^, and fat mass (FM) was determined as the difference between BM and FFM.

#### Basal metabolic rate

After overnight fasting, BMR was measured in the morning (measurements between 0800 and 1000 a.m.), using an indirect open-circuit computerized calorimetry (Vmax 29, Sensor Medics, Yorba Linda, Ca, USA) and a rigid, transparent, and ventilated canopy. Before each test, calibration was performed with a reference gas mixture (95.00% O_2_ and 5.00% CO_2_). The duration of the BMR was 45 min. Values of oxygen consumption (V′O_2_) and carbon dioxide production (V’CO_2_), standardized for temperature, barometric pressure, and humidity, were recorded at 1-min intervals. Values of the first 5–10 min were excluded from the analysis because they correspond to the adaptation to the procedure environment. Energy expenditure (EE) was calculated from the O_2_ and CO_2_ values^49^ and averaged over the entire measurement period.

#### Physical capacities and maximal fat oxidation rate

A GRAD test on a motorized treadmill determined the V’O_2_ peak values and substrate oxidation rates (TechnoGym, Gambettola, Italy). All participants performed the GRAD test in the morning (exercise starting between 0800 and 1000 a.m.), under medical supervision.

Before the start of the study, individuals were familiarized with the equipment and the procedures. All participants avoided strenuous exercise and maintained the same eating habits the day before the test and came to the laboratory after a 12-h fast.

The GRAD test began with a 10-min rest period followed by walking in stages of a 5-min duration. According to^16^ treadmill speed (m s^−1^) and incline (%) followed a sequence: 0.6 (0%), 1.0 (0%), 1.0 (3%), 1.3 (3%), 1.4 (6%), 1.4 (9%) and 1.4 (12%). The workload was gradually increased until an HR of approximately 180 beats^−1^ was reached. At this point, exercise was stopped to avoid cardiovascular complications associated with maximal effort, which would be particularly risky in this type of population. During the whole GRAD, ventilatory and gas exchange responses were measured continuously by indirect calorimetry (CPX Express, Medical Graphics Corp, MN, USA). During the exercise test, an electrocardiogram was continuously recorded and displayed online for visual monitoring whereas HR was measured with a dedicated monitor device (Polar Electro Oy, Finland). The flowmeter and gas analyzers of the system were each calibrated with a 3-L calibration syringe and calibration gas (16.00% O_2_; 4.00% CO_2_).

The V′O_2_peak was estimated for each subject considering the last 20 s of the graded exercise test.

The substrate oxidation rate was determined from V′O_2_ and V′CO_2_ values determined during the last minute of each workload level^50^ using the following equation^51^:

$${\text{Fat}}\;{\text{ oxidation}}\;{\text{ rate}}\; \, \left( {{\text{g min}}^{{ - {1}}} } \right) \, \; = \;{1}.{67 } \times {\text{ V}}^\prime {\text{O}}_{{2}} \left( {{\text{l mim}}^{{ - {1}}} } \right) \, - { 1}.{67 } \times {\text{ V}}^\prime {\text{CO}}_{{2}} \hbox{-}\left( {{\text{l mim}} - {1}} \right) \, - \, 0.{3}0{7 } \times {\text{ Pox}}$$

$${\text{Carbohydrate}}\;{\text{ oxidation}}\;{\text{ rate}}\; \, \left( {{\text{g min}}^{{ - {1}}} } \right)\; = \, \;{4}.{55 } \times {\text{ V}}^\prime {\text{CO}}_{{2}} \left( {{\text{l mim}}^{{ - {1}}} } \right) \, - { 3}.{\text{21 V}}^\prime {\text{O2 }}\left( {{\text{l mim}} - {1}} \right) \, - \, 0.{459 } \times {\text{ Pox}}$$

where Pox is the protein oxidation rate. The protein oxidation rate was estimated by assuming that protein oxidation contributed approximately 12% of resting energy expenditure^51^:

$${\text{Protein }}\;{\text{oxidation}}\;{\text{ rate}}\; \, \left( {{\text{g min}}^{{ - {1}}} } \right)\; = \;{\text{ energy}}\;{\text{ expenditure }}\;\left( {{\text{kJ min}}^{{ - {1}}} } \right) \, \; \times \; \, 0.{12} - {16}.{74 }\left( {{\text{kJ g}}^{{ - {1}}} } \right)$$

The results of the graded exercise test were used to compute the relationship between substrate oxidation and exercise intensity, expressed as %V’O_2_ peak, according to^16^. Before and after the training program, the graded exercise test was performed following the same protocol.

#### Energy expenditure and substrate oxidation rate during submaximal exercise

As described previously, all the volunteers were randomly split into two groups: 10 adolescents participated in COMB training and 11 adolescents participated in a MICT program. To ensure full recovery after the GRAD test, submaximal testing took place two days after the GRAD test. All the participants arrived at the laboratory after a 12-h overnight fast. COMB and MICT were designed to have equal amounts of energy expended. Both COMB and MICT exercises were performed on a motorized treadmill (TechnoGym, Gambettola, Italy). The COMB training test included a 10-min rest period in a standing position on a treadmill, followed by ~ 36 min of walking with 3 repetitions of 2 min at a high intensity (95% of V′O_2_ peak) followed by ~ 30 min of MICT (60% of V′O_2_ peak). The MICT exercise test comprised a 10-min rest period in a standing position on a treadmill, followed by about 45 min of walking at maximal fat oxidation rate intensity previously determined individually during a GRAD test.

During the submaximal tests, V′O_2_ and V′CO_2_ were measured continuously (CPX Express, Medical Graphics Corp, MN, USA) during the rest and exercise periods. According to Lazzer et al.^16^, the substrate oxidation rate was calculated over consecutive 5-min periods using the equations of^51^. Energy supply (kJ min^−1^) during exercise was calculated as the sum of each substrate oxidation rate (g min^−1^) multiplied by the appropriate conversion factor (carbohydrate and protein = 16.7 kJ g^−1^; fat = 37.7 kJ g^−1^). During the exercise tests an electrocardiographic record was performed continuously and displayed online for visual monitoring, and HR was measured with a dedicated monitor device (Polar Electro Oy, Finland).

### Statistical analyses

Data were analyzed using GraphPad Prism version 9.5.1 (IBM, Chicago, USA), with significance set at P < 0.05. All results are expressed as mean and standard deviation (SD). Shapiro–Wilk was used to verify the normal distribution of the data. Sphericity was verified by Mauchly’s test.

If the sphericity assumption was violated, the Greenhouse–Geisser correction was applied. To assess training adherence and the total duration of the training, unpaired Student’s t-tests were used. Anthropometric characteristics, body composition, V′O_2_ peak, training characteristics, BMR, energy expenditure, and substrate oxidation during submaximal tests were analyzed with a 2-way ANOVA or a general linear mixed model that included the between-subjects group factor (COMB or MICT) and the within-subjects time factor (week 0 *vs.* week 3, i.e., repeated-measures analysis). Post-hoc comparisons were performed using the Bonferroni procedure for significant differences. The same analyses were used for the substrate oxidation rate during the GRAD test, adding the % of V′O_2_ peak as a fixed factor to examine differences in substrate oxidation (i.e., carbohydrate and fat) rates in response to COMB or MICT training separately. A three-way ANOVA or a general linear mixed model (2 groups × 2-time points × 8 stage measurements) was conducted to examine differences in substrate oxidation rates during the test between the COMB and MICT groups. Finally, the corrected effect size (ES) was calculated for pre-post differences between COMB and MICT^52^. An ES < 0.20 was considered small, and 0.50 was considered large^53^. To estimate the sample size a priori, power analysis of 10 participants per group with an F test for repeated-measures ANOVA with a statistical power of 0.80, a probability a level of 0.05, and an effect size f of 0.45 (G-Power software, v. 3.1.9.2, Universitat Kiel, Kiel, Germany) resulted in a predicted 12% improvement in the V′O_2_^16^.

### Institutional review board statement

The study was conducted according to the guidelines of the Declaration of Helsinki and approved by the Ethics Committee of Istituto Auxologico Italiano, IRCCS, Milan, Italy (ethical committee code of approval: 2022_03_15_03; research project code: 01C212; acronym: ALPOLAROB).

### Informed consent

Informed consent was obtained from the parents of all adolescents involved in the study.

## Data Availability

Raw data will be available upon a reasonable request to the corresponding author and will be uploaded on www.zenodo.org immediately after the acceptance of the manuscript.
